# Asthma-free eosinophilic granulomatosis with polyangiitis presenting with Guillain-Barré syndrome-like symptoms: a case report

**DOI:** 10.3389/fmed.2026.1837875

**Published:** 2026-06-05

**Authors:** Haidong Huang, Xingqi Zhao, Yi Zhou, Sizhan Chen, Yanxia Zhou

**Affiliations:** 1 The First Affiliated Hospital of Shenzhen University, Shenzhen, Guangdong, China; 2 Department of Neurology, Shenzhen Second People’s Hospital, Shenzhen, China

**Keywords:** case report, eosinophilic granulomatosis with polyangiitis, Guillain-Barré syndrome, hypereosinophilia, mononeuritis multiplex

## Abstract

**Background:**

Eosinophilic granulomatosis with polyangiitis (EGPA) is a rare systemic vasculitis classically characterized by asthma, eosinophilia, and necrotizing vasculitis. Although the vast majority of EGPA patients present with concomitant asthma, this feature should not be considered an absolute prerequisite for diagnosis.

**Case report:**

A 50-years-old male presented with a 2-weeks history of progressive numbness and weakness in all four extremities, leading to an initial admission under the provisional diagnosis of Guillain-Barré syndrome (GBS). Although the subacute disease course was confounding, physical examination at our institution identified two pivotal clues: marked asymmetric involvement and severe neuropathic pain. These findings strongly suggested the possibility of vasculitic neuropathy. The patient lacked classical pulmonary manifestations, such as asthma or nasal polyposis, and pulmonary function tests were within normal limits. Nevertheless, his asymmetric mononeuritis multiplex, cutaneous rash, and marked eosinophilia (3.33 × 10^∧^9/L, 18.9%) constituted critical diagnostic evidence. A subsequent skin biopsy revealed characteristic vasculitic changes. By applying the 2022 ACR/EULAR classification criteria, the patient’s cumulative score firmly supported the diagnosis of eosinophilic granulomatosis with polyangiitis (EGPA). The patient achieved symptomatic remission following a 3-months therapeutic regimen comprising systemic glucocorticoids and mycophenolate mofetil.

**Conclusion:**

We report an exceedingly rare case of EGPA presenting with GBS-like symptoms in the absence of asthma, illustrating the remarkable clinical heterogeneity of this condition. Despite the absence of hallmark features, timely diagnosis was achieved through thorough systematic investigation, underscoring the critical importance of accurate diagnosis and prompt therapeutic intervention.

## 1 Introduction

Eosinophilic granulomatosis with polyangiitis (EGPA), formerly termed Churg-Strauss syndrome (1), is a rare systemic vasculitis with an annual incidence of approximately 1 case per million population (2, 3). The disease is classically defined by a triad of asthma, eosinophilia, and necrotizing vasculitis. Peripheral nervous system involvement is frequently observed, predominantly manifesting as mononeuritis multiplex–the hallmark neurological pattern of this condition (4, 5).

The rarity of the present case lies in its uniquely atypical clinical presentation: the patient developed acute, multifocal peripheral neuropathy bearing a striking resemblance to GBS, with no history of asthma or any atopic condition throughout the disease course. According to published literature, EGPA patients without asthma represent fewer than 5% of all cases (6), which constitutes the primary reason for the high risk of misdiagnosis as common neurological disorders such as GBS.

Differential diagnosis between EGPA and GBS is of substantial clinical importance, as the therapeutic strategies for these two conditions differ considerably. GBS is conventionally managed with intravenous immunoglobulin or plasma exchange, whereas glucocorticoids offer limited therapeutic benefit (7). In contrast, the mainstay of EGPA treatment relies on glucocorticoids and immunosuppressive agents (8); misdiagnosis may therefore result in delayed treatment and deterioration of clinical outcomes.

Herein, we report a case of asthma-free EGPA initially misdiagnosed as GBS, with the aim of raising clinical awareness regarding atypical presentations of this condition.

## 2 Case presentation

A 50-years-old male was admitted with a chief complaint of progressive numbness and weakness in all four extremities over a 2-weeks period. The illness onset was characterized by headache and right upper extremity pain, which subsequently progressed to bilateral numbness and weakness in the hands and feet. These symptoms were most pronounced in the left upper extremity and were accompanied by severe nocturnal pain, reaching approximately 7 on the Visual Analog Scale (VAS). The patient categorically denied any personal history of asthma, allergic rhinitis, or other atopic conditions.

Neurological examination revealed an asymmetric pattern of muscle weakness: proximal motor strength in the bilateral upper extremities was grade 5/5, whereas distal strength was grade 3/5 on the left and grade 4/5 on the right, accompanied by markedly diminished bilateral grip strength. In the lower extremities, motor strength was grade 4/5 on the left and grade 5/5 on the right. Hypoalgesia and diminished tactile sensation were noted distally below the wrists and ankles bilaterally.

Based on the clinical presentation and the provisional diagnosis from the referring hospital, Guillain-Barré syndrome (GBS) was initially considered upon admission. However, a comprehensive clinical evaluation revealed several critical findings inconsistent with GBS. First, the patient exhibited a markedly asymmetric pattern of neurological deficit, which contradicts the classic symmetric involvement typical of GBS. Second, the clinical picture was complicated by intense, electric-shock-like neuropathic pain (VAS score of 7), whereas classic GBS generally presents with minimal pain or mild sensory abnormalities. Third, cerebrospinal fluid (CSF) analysis demonstrated normal opening pressure, cell count, and protein levels, completely lacking the albuminocytologic dissociation that is the hallmark of GBS.

**FIGURE 1:**
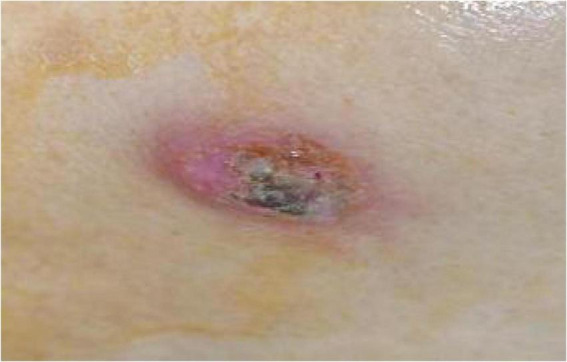
Skin rash over the patient’s right shoulder.

These clinical discrepancies prompted further investigation into underlying systemic inflammatory etiologies. A supplementary physical examination revealed scattered erythematous nodular skin lesions with central pustulation over the bilateral shoulders (Figure 1). A detailed summary of the laboratory and imaging investigations is provided in Table 1.

**TABLE 1:** Laboratory and imaging findings.

Examination	Result	Reference range
White blood cell count (WBC)	17.64 × 10^9^/L ↑	3.5–9.5 × 10^9^/L
Eosinophil count/percentage	3.33 × 10^9^/L (18.9%) ↑	0.02–0.5 × 10^9^/L (<5%)
Erythrocyte sedimentation rate (ESR)	64.94 mm/h ↑	<15 mm/h
High-sensitivity C-reactive protein (hs-CRP)	46.09 mg/L ↑	<5 mg/L
Total IgE	>6000.0 ng/mL ↑	0–150 ng/mL
Interleukin-6 (IL-6)	29.88 pg/mL ↑	<7 pg/mL
ANCA (MPO/PR3)	Negative	–
Bone marrow biopsy	Eosinophils 10.07%; no blasts identified	–
Cerebrospinal fluid (CSF)	Pressure, cell count, and protein within normal limits	–
Pulmonary function test	No obstructive ventilatory impairment (FEV1/FVC ≥ 70%)	–
Chest CT/cranial MRI/cervical MRI	No significant abnormalities	–

The upward arrows “↑” denote values above the upper limit of the normal reference range.

In addition, a full anti-ganglioside antibody panel (anti-GM1, GM2, GM3, GD1a, GD1b, GD2, GD3, and GQ1b), antinuclear antibody (ANA), extractable nuclear antigen (ENA), anticardiolipin antibody (ACA), and a comprehensive parasite antibody panel were all negative, with no blasts identified on bone marrow biopsy.

Nerve conduction studies clearly demonstrated an asymmetric, multifocal, mixed (axonal and demyelinating) peripheral neuropathy (Table 2). The predominantly affected nerves included the median, ulnar, common peroneal, and tibial nerves. No identifiable waveforms could be elicited from the left median nerve in both motor and sensory modalities, while the right median nerve and bilateral lower limb nerves were affected to varying degrees, displaying a characteristically asymmetric and multifocal distribution highly suggestive of mononeuritis multiplex rather than diffuse polyneuropathy. Bilateral radial nerve motor and sensory conduction studies were within normal limits. Needle electromyography of the examined muscles (bilateral tibialis anterior, gastrocnemius, abductor pollicis brevis, abductor digiti minimi, etc.) revealed no spontaneous activity, and motor unit potential duration, amplitude, and polyphasic wave proportions were all within normal ranges, indicating no evidence of active denervation at the time of examination.

**TABLE 2:** Nerve conduction study results.

Motor nerve conduction				
**Nerve**	**Segment**	**Latency (ms)**	**Amplitude (mV)**	**Conduction velocity (m/s)**
Left median nerve	Wrist	NA	NA	NA
	Elbow→wrist	NA	NA	NA
Right median nerve	Wrist	3.05	8.09	32.8
	Elbow→wrist	7.45	1.34↓	50.0
Left ulnar nerve	Wrist	2.80	0.09↓	21.4
	Elbow→wrist	7.95	0.37↓	44.7
Right ulnar nerve	Wrist	3.30	0.92↓	21.2
	Elbow→wrist	8.15	1.07↓	47.1
Left common peroneal nerve	Anterior ankle	3.85	2.33	22.1
	Fibular head→anterior ankle	10.1	1.21↓	48.0
Right common peroneal nerve	Anterior ankle	4.30	2.67	18.6
	Fibular head→anterior ankle	10.9	2.13↓	45.5
Left tibial nerve	Medial malleolus	6.60	2.74	19.7
	Popliteal fossa→medial malleolus	15.1	7.94	48.0
Right tibial nerve	Medial malleolus	6.15	1.79↓	19.5
	Popliteal fossa→medial malleolus	15.2	2.98↓	12.0
Sensory nerve conduction				
**Nerve**	**Stimulation/recording site**	**Latency (ms)**	**Amplitude (μV)**	**Conduction velocity (m/s)**
Left median nerve	Middle finger/wrist	NA	NA	NA
Right median nerve	Middle finger/wrist	2.80	4.47	55.4
Left ulnar nerve	Little finger/wrist	2.40	0.27↓	54.2
Right ulnar nerve	Little finger/wrist	4.33↑	5.08	27.7↓
Left sural nerve	11 cm above calcaneus/lateral malleolus	2.33	2.81↓	66.4
Right sural nerve	14 cm above calcaneus/lateral malleolus	2.47	13.2	60.8
Left superficial peroneal nerve	Peroneus longus/dorsum of foot	–	6.21	46.7
Right superficial peroneal nerve	Peroneus longus/dorsum of foot	NA	NA	NA

NA = No identifiable waveform; ↓ = Below the lower limit of normal; ↑ = Above the upper limit of normal. F-wave studies demonstrated reduced occurrence rates and prolonged latencies in most affected nerves, consistent with the distal axonal involvement described above, and are not listed separately. Bilateral radial nerve conduction and needle EMG findings were within normal limits and are not listed separately.

**FIGURE 2:**
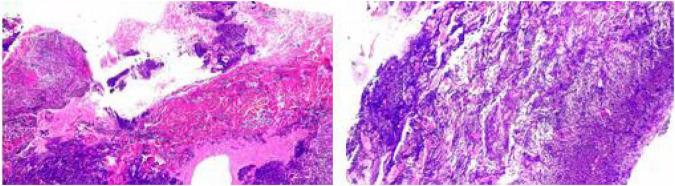
Skin biopsy (H&E staining).

The other critical investigation was histopathological examination. Skin biopsy of the right shoulder lesion demonstrated fibrovascular proliferation with mixed acute and chronic inflammatory cell infiltration; abundant interstitial infiltration of lymphocytes, neutrophils, and eosinophils with abscess formation was identified, consistent with the diagnosis of vasculitis (Figure 2).

The 2022 ACR/EULAR Classification Criteria for Eosinophilic Granulomatosis with Polyangiitis (9) were applied for scoring: a maximum eosinophil count ≥ 1 × 10^9^/L scored + 5 points, and mononeuritis multiplex not attributable to radiculopathy scored + 1 point, yielding a total score of 6 points. According to this classification system, a score ≥ 6 is sufficient to classify the condition as EGPA (Table 3). Given that this classification system demonstrates 85% sensitivity and 99% specificity when applied to patients with confirmed vasculitis, the patient was ultimately diagnosed with ANCA-negative eosinophilic granulomatosis with polyangiitis (EGPA).

**TABLE 3:** 2022 ACR/EULAR classification criteria for eosinophilic granulomatosis with polyangiitis and scoring for the present case.

Clinical criterion	Points	Present case
Obstructive airway disease	+3	No (0 points)
Nasal polyps	+3	No (0 points)
Mononeuritis/motor neuropathy not due to radiculopathy	+1	Yes (1 point)
Blood eosinophil count ≥ 1 × 10^9^/L	+5	Yes, 3.33 × 10^9^/L (5 points)
Extravascular eosinophil-predominant inflammation	+2	No (0 points)
c-ANCA or anti-PR3-ANCA positivity	−3	No (0 points)
Hematuria	−1	No (0 points)
Total score		6 points

**FIGURE 3:**
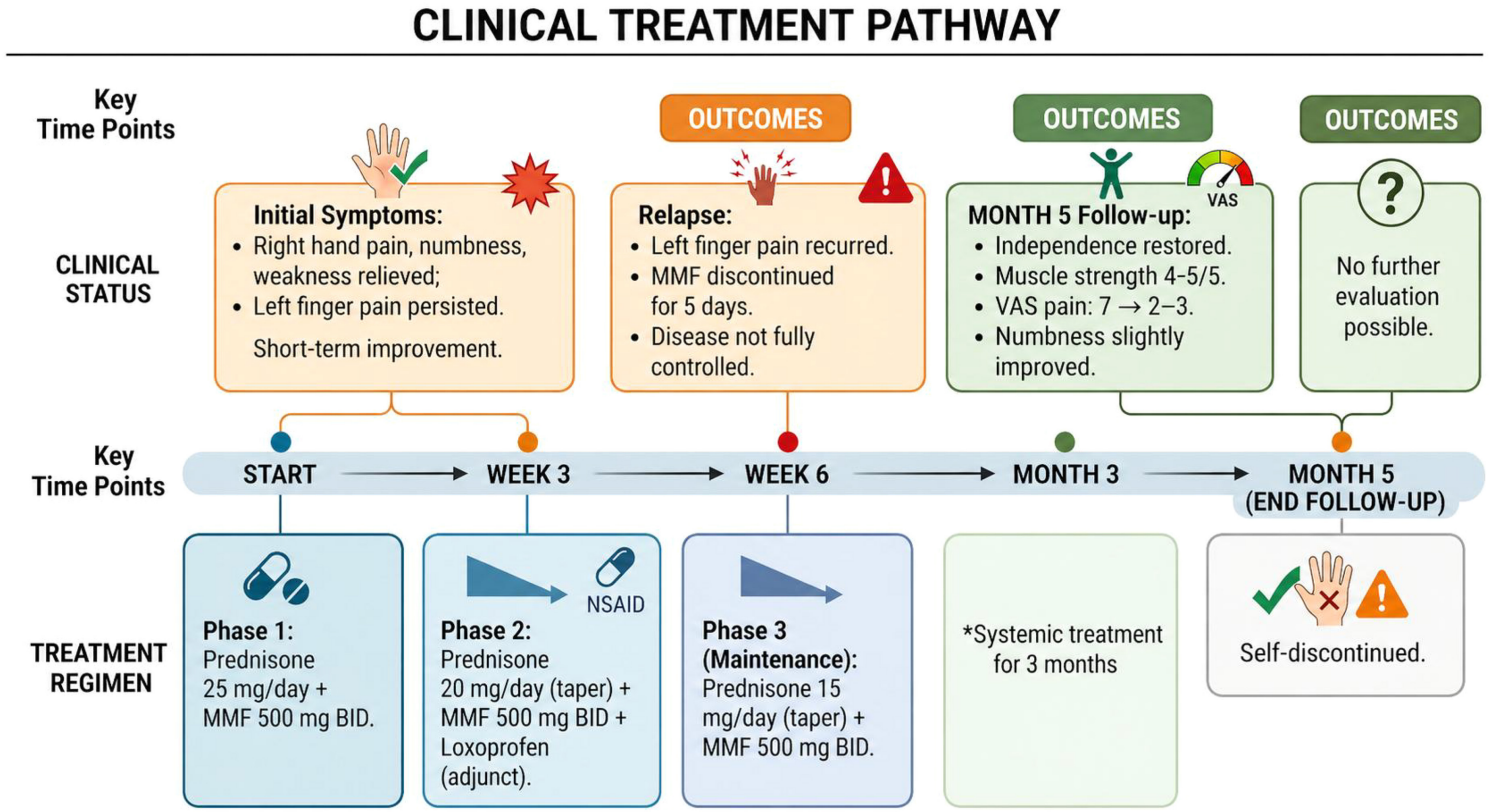
Timeline of clinical management and treatment response. “*” Highlights that systemic treatment was limited to 3 months due to patient self-discontinuation, which prevented long-term efficacy assessment.

Following a definitive diagnosis, the patient was initiated on a comprehensive treatment regimen consisting of prednisone 25 mg/day and mycophenolate mofetil 500 mg twice daily. Initial therapy resulted in marked alleviation of pain, numbness, and weakness in the right hand. However, relief of the left finger joint pain was less pronounced, indicating a partial short-term clinical remission. Given the overall significant clinical improvement, the prednisone dose was tapered to 20 mg/day at week 3 of treatment to minimize corticosteroid-related adverse effects and facilitate the transition to maintenance therapy. Loxoprofen sodium was concurrently added as an adjuvant non-steroidal anti-inflammatory treatment. The patient was reassessed at week 6 of treatment; at that time, he had self-discontinued mycophenolate mofetil for 5 days and presented with recurrent pain in his left finger joints, suggesting incomplete disease control. Consequently, treatment was continued with prednisone further tapered to 15 mg/day alongside mycophenolate mofetil 500 mg twice daily as maintenance therapy. At the 5-months follow-up, the patient had regained the ability to work and perform daily activities independently. Motor strength had progressively recovered to grade 4–5, the Visual Analog Scale (VAS) pain score had decreased substantially from 7 to 2–3, and limb numbness showed modest improvement. However, it was noted that the patient had self-discontinued both systemic glucocorticoids and the mycophenolate mofetil immunomodulatory regimen after 3 months of treatment, precluding further evaluation of long-term therapeutic efficacy. The clinical treatment pathway is summarized in Figure 3. This case highlights that in the long-term management of rare conditions such as EGPA, enhancing patient education regarding the disease and strictly managing medication adherence are of paramount importance.

## 3 Discussion

Eosinophilic granulomatosis with polyangiitis, also known as Churg-Strauss syndrome, is a rare multisystem vasculitis typically characterized by asthma, necrotizing vasculitis, and prominent peripheral eosinophilia. The pathogenesis of EGPA has not been fully elucidated; its clinical course can be divided into an allergic phase, an eosinophilic phase, and a vasculitic phase, with overlapping manifestations across phases, though not all patients progress through the complete three-stage course. The present case, characterized by the exceedingly rare combination of absent asthma and a GBS-like onset, provides valuable insights into the atypical clinical spectrum of EGPA.

The immunopathogenesis of EGPA is centered on a Th2-predominant immune response, in which cytokines including IL-4, IL-5, and IL-13 drive eosinophil proliferation and activation. Activated eosinophils infiltrate vascular walls and release granule proteins, inducing small vessel necrosis, tissue ischemia, and granuloma formation, ultimately resulting in multiorgan damage (10, 11). The markedly elevated IgE level in this patient (>6,000 ng/mL) is consistent with the mechanism of excessive Th2 immune activation described above. Furthermore, activated eosinophils produce a variety of pro-inflammatory mediators that indirectly promote the release of cytokines such as IL-6 (12, 13). Elevated IL-6 is transported via the bloodstream to the liver, where it rapidly induces the synthesis of acute-phase proteins including C-reactive protein (CRP) and fibrinogen (14); CRP directly reflects the degree of inflammatory activity, while elevated fibrinogen promotes erythrocyte aggregation and accelerates the erythrocyte sedimentation rate (ESR) (15). The markedly elevated IL-6 (29.88 pg/mL), hs-CRP (46.09 mg/L), and ESR (64.94 mm/h) observed in this patient are consistent with published reports indicating that CRP and ESR are significantly elevated in more than 50% of patients during active EGPA (16).

Driven by the aforementioned immunoinflammatory mechanisms, the present case exhibited three atypical clinical features that rendered the diagnostic process particularly challenging. The rarity of this case is concentrated in the following three aspects. First, this case completely lacked asthma–the hallmark feature of EGPA. Published literature reports that more than 96% of EGPA patients have concomitant asthma, with asthma-free patients accounting for fewer than 5% of cases (6, 17), which is the fundamental reason why this case was highly susceptible to diagnostic error. Second, this case presented with multifocal peripheral neuropathy as the initial manifestation, with a subacute onset and prominent acral pain mimicking Guillain-Barré syndrome–an unusual mode of presentation that has been documented in only a limited number of case reports (18). Early diagnostic confusion is common because both conditions can exhibit a subacute progressive course. Pathophysiologically, however, mononeuritis multiplex in EGPA results from ischemic nerve infarction caused by the occlusion of the vasa nervorum, which clinically manifests as severe axonal damage and asymmetric involvement. In contrast, GBS is classically characterized by immune-mediated, symmetric demyelination. Electrophysiological studies in such vasculitic neuropathies typically reveal a marked reduction in nerve action potential amplitude (19). This underlying ischemic mechanism directly elucidates the pathological basis of the mononeuritis multiplex observed in the present case. Additionally, cutaneous involvement occurs in approximately 50% of EGPA patients, with diverse manifestations; purpura is the most common presentation (25%), followed by urticarial-like rash and subcutaneous nodules, while gangrenous and necrotic lesions are relatively uncommon (6, 20, 21). The scattered erythematous nodular skin lesions over bilateral shoulders and the necrotizing vasculitis identified on skin biopsy in this case represent a typical manifestation of EGPA cutaneous involvement, and constituted one of the key pathological bases for the definitive diagnosis. Notably, although eosinophilic infiltration was identified on skin biopsy in this case, the pattern of infiltration was a mixed distribution of lymphocytes, neutrophils, and eosinophils rather than the “predominantly extravascular eosinophilic inflammation” described in the classical pathology of EGPA; consequently, the +2 points for this criterion within the ACR/EULAR classification system were not awarded. This finding may be attributable to the fact that the patient was in the vasculitic phase at the time of biopsy–with the eosinophil-predominant infiltration having already subsided–as well as prior short-term corticosteroid use accelerating eosinophil apoptosis within the tissue (21, 22). This observation further serves as a reminder to clinicians that the absence of typical eosinophilic infiltration on skin biopsy does not exclude EGPA; a comprehensive assessment incorporating peripheral blood eosinophil counts and systemic clinical manifestations remains essential.

Following the characterization of these three atypical features, systematic etiological investigation and application of classification criteria were pivotal to achieving the definitive diagnosis. The etiological spectrum of hypereosinophilia is broad, encompassing parasitic infections, hematologic malignancies (such as eosinophilic leukemia), and other connective tissue diseases. First and foremost, it is imperative to distinguish EGPA from hypereosinophilic syndrome (HES). Although both conditions are characterized by marked eosinophilia, HES typically presents with multisystem eosinophilic infiltrative damage and lacks histological evidence of vasculitis. In the present case, the skin biopsy unequivocally demonstrated vasculitic changes, and the diagnosis of EGPA was firmly established based on the ACR/EULAR classification criteria. Furthermore, we systematically excluded other common causes of eosinophilia: (1) Parasitic infections: These were ruled out by a negative comprehensive parasite antibody panel. (2) Hematologic malignancies: Bone marrow aspiration revealed a mildly elevated proportion of eosinophils but no blasts, thereby excluding chronic eosinophilic leukemia (CEL). (3) Other ANCA-associated vasculitides (AAV): Conditions such as granulomatosis with polyangiitis (GPA) and microscopic polyangiitis (MPA) frequently involve the peripheral nervous system, but they are generally not associated with such prominent eosinophilia. On this basis, application of the 2022 ACR/EULAR classification criteria yielded a score of 6 points–blood eosinophil count ≥ 1 × 10^9^/L (+5 points) and mononeuritis multiplex (+1 point)–confirming the diagnosis of EGPA (9).

Regarding treatment, the therapeutic strategies for EGPA and GBS differ fundamentally, which underscores the core significance of early and accurate differential diagnosis. Glucocorticoids constitute the cornerstone of EGPA therapy, and the overall treatment strategy is conducted in two phases (8, 10): the remission induction phase employs glucocorticoids and/or immunosuppressive agents, while the maintenance phase favors azathioprine or methotrexate. Given the chronic relapsing nature of the disease and the need to balance efficacy against adverse effects such as myelosuppression, mycophenolate mofetil was selected as the immunosuppressive agent in this case. Following treatment with prednisone combined with mycophenolate mofetil, the patient achieved marked symptomatic improvement, with the VAS pain score decreasing from 7 to 2–3 and muscle strength progressively recovering to grade 4–5, confirming the critical importance of early accurate diagnosis and timely therapeutic intervention.

## 4 Conclusion

In summary, this case underscores that peripheral blood eosinophil count represents a straightforward yet pivotal differential diagnostic indicator when evaluating patients with peripheral neuropathy. Even in the absence of a history of asthma, the detection of markedly elevated eosinophil counts should prompt a high degree of clinical suspicion for EGPA. A systematic assessment integrating nerve conduction studies, pathological biopsy, and the 2022 ACR/EULAR classification criteria is essential to avoid misdiagnosis and missed diagnosis, thereby ensuring optimal timing for therapeutic intervention.

## Data Availability

The original contributions presented in this study are included in this article/supplementary material, further inquiries can be directed to the corresponding authors.
